# Extracellular BMP Antagonists, Multifaceted Orchestrators in the Tumor and Its Microenvironment

**DOI:** 10.3390/ijms21113888

**Published:** 2020-05-29

**Authors:** Sarah Ouahoud, James C.H. Hardwick, Lukas J.A.C. Hawinkels

**Affiliations:** Department of Gastroenterology and Hepatology, Leiden University Medical Centre, 2333 ZA Leiden, The Netherlands; S.ouahoud@lumc.nl (S.O.); J.C.H.Hardwick@lumc.nl (J.C.H.H.)

**Keywords:** bone morphogenetic proteins, antagonists, Noggin, Gremlin, tumor microenvironment, cancer

## Abstract

The bone morphogenetic proteins (BMPs), a subgroup of the transforming growth factor-β (TGF-β) superfamily, are involved in multiple biological processes such as embryonic development and maintenance of adult tissue homeostasis. The importance of a functional BMP pathway is underlined by various diseases, including cancer, which can arise as a consequence of dysregulated BMP signaling. Mutations in crucial elements of this signaling pathway, such as receptors, have been reported to disrupt BMP signaling. Next to that, aberrant expression of BMP antagonists could also contribute to abrogated signaling. In this review we set out to highlight how BMP antagonists affect not only the cancer cells, but also the other cells present in the microenvironment to influence cancer progression.

## 1. Introduction

The bone morphogenetic proteins (BMPs) belong to the transforming growth factor (TGF)-β superfamily, which also comprises the TGF-βs, activins, nodal, inhibins and myostatin [1]. While BMPs were first discovered because of their ability to promote endochondral bone growth, hence the name, BMP action is now known to contribute to several crucial biological processes throughout the entire body ranging from embryonic development and patterning to adult tissue homeostasis and control of stem cells and their niches [2]. Since BMPs are implicated in such diverse biological processes, it has been suggested that their name should be changed to body morphogenic proteins [2].

**Figure 1:**
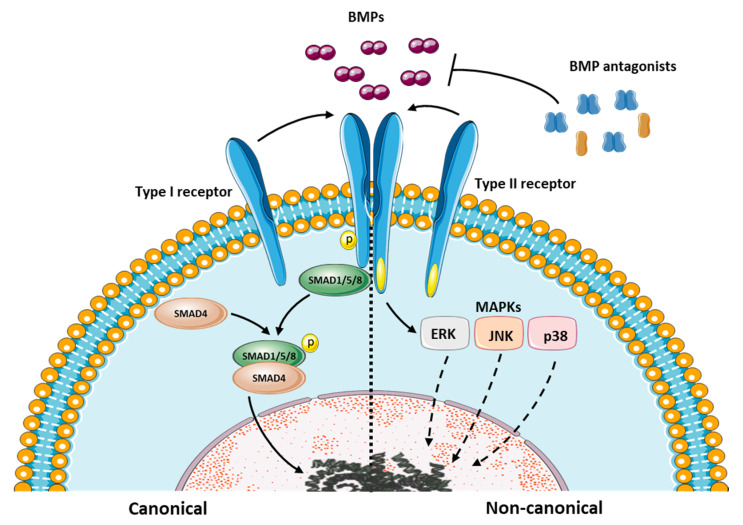
A schematic representation of the bone morphogenetic proteins (BMP) signaling cascade. BMP antagonists are important regulators of BMP signaling amplitude as they directly bind BMPs, thereby preventing them from interacting with the receptors.

Canonical BMP signaling (Figure 1) takes places when BMP ligands interact with the type I and type II BMP receptors, inducing heteromeric complex formation of the two different receptor types. The constitutively active type II receptors then phosphorylate the type I receptors, which phosphorylate the SMAD proteins SMAD1, SMAD5 and SMAD8 [3]. The activated SMAD complex binds SMAD4 after which it is translocated to the nucleus where they regulate transcription of BMP target genes. In addition, non-canonical BMP signaling occurs via mitogen-activated protein kinases (MAPKs) in a SMAD-independent manner (Figure 1) [3]. BMP pathway activity is dependent on tissue specific BMP ligand expression and the presence of BMP receptors on the cells [4]. Additionally, local BMP bioavailability and subsequent BMP signaling is further regulated by a group of molecules which bind and sequester BMPs, collectively called BMP antagonists. By binding to the BMPs, the BMP ligands can no longer bind to their receptor and BMP signaling is prevented [5]. These interactions between the BMPs and their respective antagonists are necessary to govern the BMP signaling amplitude needed for the biological processes to take place successfully [6]. In vivo, the outcome of BMP signaling in relation to the BMP antagonists is complex. Some antagonists have been shown to inhibit BMPs when present in high concentrations, while stimulating BMP activity when present at low concentrations [4]. Besides interacting with BMPs, the antagonists have also been shown to interact with one another. The binding of one antagonist to another type of antagonist can potentiate the effect of the antagonist or inhibit it. To make antagonist-mediated BMP signaling even more complex, there is interplay with several other signaling pathways such as Wnt, Notch, Sonic hedgehog (Shh) and the fibroblast growth factor (FGF) pathway [5]. Additionally, besides directly inhibiting BMP signaling, BMP antagonists have also been shown to elicit their effect through modulation of these other pathways [7]. This makes the outcome of BMP signaling an intricate process that is highly dependent on the cellular context.

## 2. Classification of BMP Antagonists

**Figure 2:**
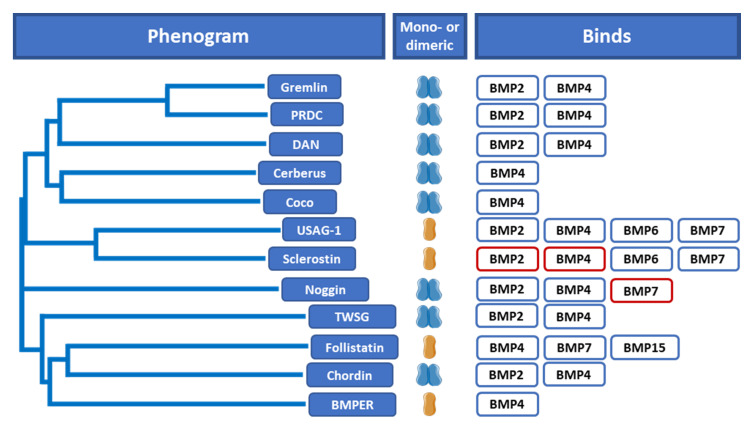
Based on the amino acid sequence, the antagonists do not share significant sequence similarities and the cystine-knots are considered to be the defining feature for most antagonists. While many of the antagonists form dimers, USAG-1, Sclerostin, Follistatin and BMPER are secreted as monomers. The different antagonists bind various BMP ligands. BMPs that have been reported to form weak interactions with an antagonist are shown in red. (This figure only shows the most well-known and studied antagonists).

Presently, multiple BMP antagonists have been reported. No significant similarities are found when amino acid sequences are compared (Figure 2). That the antagonists all belong to a single family becomes more clear towards the C terminus or cystine-knot domain of the proteins [8]. Most BMP antagonists are subclassified into subgroups based on their cystine-knot size [9]. These cystine-knots are functional motifs that determine how the peptides are folded and which hydrophobic residues, needed for protein–protein interaction, are exposed [10]. In the subclassification system based on the cystine-knot size, most of the antagonists are categorized in three main subgroups. The differential screening selected gene aberrative in neuroblastoma (DAN) subfamily, consisting of DAN, the Cerberus homologue Cer1, Coco, protein related to Dan and Cerberus (PRDC), Gremlin, uterine sensitization-associated gene 1 (USAG-1) and Sclerostin, possess a cystine-knot with eight cysteine residues making up the ring of the knot (Figure 2) [9]. The second subgroup consists of twisted gastrulation (TSG) only which has a nine-cysteine ring. Chordin and Noggin make up the third subgroup and have ten cysteine residues in the cystine-knot [9]. Many of the antagonists form homodimers but some are reported to be monomers (Figure 2) [4].

## 3. BMP Antagonists and Cancer

In adult tissue, it is increasingly acknowledged that the subversion of the balance between BMPs and their antagonists may underlie several diseases, including cancer. To understand how BMP antagonists could contribute to oncogenesis, some background on how BMP agonists exert their function is needed. BMPs are thought to play a tumor-suppressing role as BMPs induce cell differentiation and apoptosis and therefore loss of a crucial signaling component could result in increased cell proliferation [11]. However, it seems like their role, tumor promoting or tumor suppressing, depends on the specific BMP ligand, the cancer type and the tumor stage. Multiple studies, both in animal models and in humans, have indeed demonstrated a strong relationship between epithelial loss of functional BMP receptors and the initiation or progression of specific cancers [12]. Theoretically, BMP antagonists could be expected to play a tumor-promoting role as BMPs induce cell differentiation and apoptosis and BMP antagonists could inhibit BMPs from doing so. The BMP pathway has been implicated in various stages of carcinogenesis in multiple cancers [13]. Many studies have reported involvement of the pathway in the proliferation, migration and invasion of epithelial cancer cells. Next to increased expression of matrix metalloproteinases and integrins, which contribute to the increased migration and invasion capacity of the cancerous cells, BMPs have also been shown to induce epithelial to mesenchymal transition [13]. These are all processes that help the tumor cells to successfully metastasize.

A very illustrative example of how deregulation of BMP signaling could contribute to carcinogenesis can be found in the intestines. The intestines are well known for their high cellular turnover [14]. In humans, it takes around 4–7 days to completely replenish the epithelial cells composing the crypt-villus axis [15]. Cell renewal is tightly regulated by various signaling pathways with the Wnt and BMP pathway being key players [15,16]. While Wnt signaling drives cell proliferation of the stem cells and transient amplifying cells in the crypts, BMP signaling becomes more prominent in the top part of the crypt-villus axis. Here it makes sure that cells differentiate and commit to a certain cell lineage, thereby losing their proliferative features [15,16]. Individuals with juvenile polyposis syndrome (JPS) are carriers of mutations in crucial components of the BMP pathway such as the BMP receptor 1a (BMPR1a) or the downstream signaling molecule SMAD4. Individuals carrying these mutations develop multiple polyps throughout the gastrointestinal tract from a very young age. The discovery of the relationship between the loss of BMP pathway components and JPS led to the consideration that BMP inactivation could be involved in sporadic colorectal cancers (CRC) as well. Indeed, the BMP pathway was found to be abrogated in a large number of sporadic CRC cases [17].

In the same way, dysregulation of BMP antagonists may likewise contribute to oncogenesis. An in vivo study with transgenic mice overexpressing Noggin showed that overexpression resulted in a loss of the normal crypt-villus architecture along with de novo crypt formation and neoplasia. This is probably due to Noggin antagonizing BMP signaling and therefore inhibiting differentiation and apoptosis of epithelial cells. Interestingly, the authors state that the intestinal changes in these mice phenocopy the histopathology seen in intestines of patients with JPS [18]. These data illustrate how the overexpression of a BMP antagonist can result in a similar outcome compared to when a crucial factor required for BMP signaling is lost. In practice, upregulation of a BMP antagonist to overcome BMP signaling is rarely seen in cancer. Most studies have rather reported downregulation of BMP antagonists.

## 4. The Tumor and Its Microenvironment

**Figure 3:**
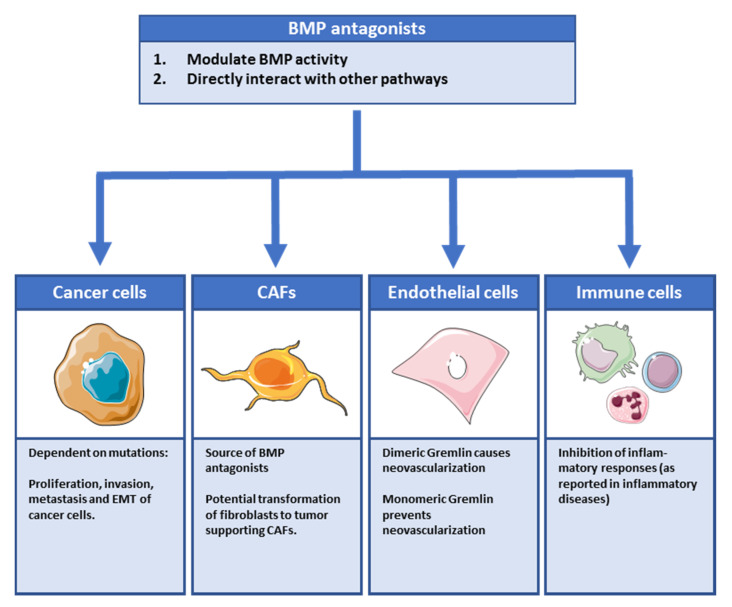
Overview of effects of BMP antagonists on cells in the tumor microenvironment. Regarding effects of BMP antagonists, many studies with conflicting data have been reported. There are multiple ways by which BMP antagonists can influence cancer progression as different cell types, apart from the cancer cells, can be affected.

While a relatively large amount of attention has been given to aberrant BMP signaling in cancer cells, very little attention has been given to the other cells present within the tumor. The role of the tumor microenvironment is increasingly receiving recognition in cancer progression. The bidirectional exchange of information between epithelial cells and their microenvironment is not only crucial for the maintenance of adult tissue homeostasis but also determines the rate and aggressiveness with which cancers progress. For example, our group recently demonstrated that fibroblasts upregulate BMP2 as a reaction to tumor secreted tumor necrosis factor-related apoptosis inducing ligand (TRAIL) [19]. Fibroblast secreted BMP2 in turn stimulated migration, invasion and metastasis formation in the liver. The impact of non-epithelial parts of the tumor, also called “stroma” on cancer initiation and development can no longer be denied as studies suggest that stroma even has the ability to “normalize” aggressive oncogenic mutations in epithelial cells to such an extent that these cells will not evolve into a tumor [20,21,22]. The cancer associated fibroblasts (CAFs), endothelial cells and immune cells that compose the stromal compartment, all respond to or secrete BMP antagonists (Figure 3). In the sections below, we will discuss some of the main findings per cell type and how they are affected by BMP antagonists. It is noteworthy that although many BMP antagonists have been identified, only a few are being researched in the context of cancer: Noggin, Gremlin and, to a lesser extent, Chordin, Sclerostin and PRDC.

### 4.1. The Cancer Cells

It is well acknowledged that carcinomas are formed due to an accumulation of mutations in cells of epithelial origin [23]. These mutations allow cells to proliferate rapidly. Additional mutations, both on genetic and epigenetic level, in these cancer cells drive tumor progression as new traits are acquired that cause the tumor to behave more aggressively. Multiple studies, both in animal models and in humans, have demonstrated a strong relationship between the loss of functional BMP receptors and the initiation or progression of specific cancers [12]. However, when it comes to the role of BMP antagonists in cancer progression, conflicting results have been reported. While some studies reported that these antagonists have a growth inhibiting effect and are upregulated in some cancers, others showed the opposite. An overview of these studies can be found in Table 1.

#### 4.1.1. Gremlin

Despite these conflicting data there are some good indications that BMP antagonists are involved in cancer progression. The most compelling evidence for the role of a BMP antagonist, Gremlin in this case, in human cancer development comes from a study in which a 40 kb duplication upstream *GREM1* was analyzed. This duplication was found in a large family of Ashkenazi Jews suffering from hereditary mixed polyposis syndrome (HMPS) and the *GREM1* locus was attributed to be causal for the histopathology [24]. Additionally, transcription enhancer elements encoded by genes present within this duplication found in HMPS patients were shown to interact in vivo with the *GREM1* promotor to further enhance gene expression [24]. Multiple *GREM1* duplications have been reported since and overexpression of *GREM1* is thought to lead to polyp formation and cancer in the intestine [25]. This seems to occur via the formation of ectopic crypts thereby distorting the normal crypt-villus architecture. This presumably exposes stem cells within the ectopic crypts to the toxic environment outside the true crypt-base, thereby predisposing them to cancer. The stem cell niche seems to be defined by high levels of *GREM1* that are normally only expressed by the pericryptal fibroblasts. Ectopic expression of high levels of *GREM1* by epithelial cells in the villus leads to cells halfway up the villus behaving as stem cells and forming ectopic crypts. *GREM1* overexpression could therefore create an environment that allows for maintenance of stemness and an increase in the number of cells with the ability to proliferate.

#### 4.1.2. Noggin

The link between Noggin and cancer has been mainly investigated in cancers metastasizing to the bone. It has been shown that prostate and non-small cell lung cancer (NSCLC) cell lines overexpressing Noggin show decreased growth/expansion capabilities in a xenograft mouse model [26,27]. In addition, several in vitro studies have found Noggin to be downregulated in cancer cell lines of different origin and it has the ability to counteract the tumorigenic processes initiated by BMPs, for example, proliferation, migration, etcetera [28,29,30,31,32]. Although these functional studies provide important information regarding the role of Noggin in tumor progression, it should be noted that the models employed a non-endogenous Noggin overexpression approach and do not provide direct evidence that these mechanisms are also exploited by mammalian (cancer) cells.

#### 4.1.3. Others

Compared to Noggin and Gremlin, substantially less research has been conducted on the role of Chordin and Sclerostin. Chordin was found to be downregulated in ovarian tumors compared to both normal tissue and, more specifically, the epithelial lining covering the surface of the ovaries. It was further shown that re-expression of Chordin in ovarian cancer cell lines decreased migration and invasion [33]. Sclerostin, encoded by the sclerostin domain-containing protein 1 (*SOSTDC1*) gene, was recently found to be negatively correlated with the aggressiveness of non-small cell lung cancer and gastric cancer as lower expression was observed in metastases compared to primary tumors [34,35]. *PRDC*, a *GREM1* homologue showing strong resemblance to Noggin and Chordin as well, was recently connected to cancer progression [36]. *PRDC* was found to be downregulated in a microarray gene expression analysis performed on five endometrial cancer (EC) specimens compared to normal leiomyoma tissue. In addition, the presence of PRDC was found to inhibit proliferation of the EC cancer cell lines Ishikawa and HEC-1A in a dose dependent manner [37]. An opposing role for PRDC in cancer progression was reported in a study on gastric cancer, where it was shown to be upregulated. Silencing of *PRDC* resulted in decreased proliferation, migration and invasion in vitro while preventing tumor formation and lymph node metastasis in vivo [38]. Taken together the role of PRDC in tumor progression remains unclear.

The conflicting data, when it comes to the effect of BMP antagonists on cancer cells, could be partially explained by the genetic makeup of the cancer cells. At first it seems beneficial for cancer cells to inactivate the BMP pathway to render them non-susceptible to differentiation and apoptosis. However, several BMPs such as BMP2 and BMP4, have been found to be upregulated in multiple cancers where they contribute to migration, invasion and dissemination [19,39,40,41]. While canonical BMP signaling results in cell differentiation and apoptosis, non-canonical (non-SMAD4) signaling leads to activation of phosphoinositide 3-kinases (PI3Ks), MAPKs and the Ras homolog (Rho) family of GTPases [13]. These pathways are generally linked to the induction of angiogenesis, cell proliferation, cell survival and metastasis in various cancer types [31]. So, while cancer cells would generally benefit from abrogating BMP signaling, cancer cells with a non-functional canonical pathway (e.g., due to loss of SMAD4) could profit from active BMP signaling. Indeed, some studies have shown that BMP signaling changes from tumor suppressing to tumor promoting upon loss of SMAD4 [41,42,43]. Therefore, the genetic makeup, or mutanome of the cancer cells, seems to add a layer of complexity to an already complex topic.

A second layer of complexity is the BMP antagonists themselves. We could speculate that the effect of the antagonists will be dependent on the mutation profile as well, so that cancer cells that downregulate the antagonists could also be the cancer cells that flourish in the presence of BMPs due to non-canonical signaling. The cancer cells that upregulate the BMP antagonists (by themselves or by instructing the microenvironment) could be the cells with intact canonical BMP signaling. These are, unfortunately, questions we cannot answer yet due to the complexity of processes involved in tumor development and the heterogeneity between cancer types and the different cancer cell lines. These ideas also assume that the BMP antagonists carry out their effect exclusively via the sequestering of BMP ligands. While this could indeed be true for many of the studies, angiogenesis resulting from the binding of Gremlin to vascular endothelial growth factor (VEGF) receptor 2 (discussed later), proves that this is not necessarily the case. It would be very valuable if researchers in the future could ascertain whether BMP antagonists act via their effect on sequestering BMPs or independent from them. This could greatly increase our insights into the role of BMP antagonists.

### 4.2. The Cancer Associated Fibroblasts

A major constituent of the stroma are CAFs that arise from normal resident fibroblasts that become “activated” by cytokines in the tumor microenvironment. Furthermore, it is thought that CAFs can also be derived from bone marrow-derived mesenchymal cells, epithelial to mesenchymal transition (EMT) or smooth muscle cells/pericytes from the vasculature. During recent years, the enormous heterogeneity within the phenotype and function of the CAF population has been increasingly unveiled. While CAFs were thought to always express α-smooth muscle actin (α-SMA), fibroblast activation protein (FAP), platelet-derived growth factor (PDGF) receptor α/β and cluster of differentiation 90 (CD90), recently subsets of CAFs negative for these markers have been identified, which execute different roles (e.g., in cancer, inflammation and homeostasis) [44]. It is only recently that a consensus on the nomenclature and functioning of CAFs has been proposed [45]. Below we will discuss the studies regarding BMP antagonists and fibroblasts. Since fibroblasts are the largest stromal constituent, this section will also include studies in which the total stroma was studied. If not clearly stated in the study, we will refer to fibroblasts when explicitly mentioned and refer to stroma when insufficiently defined.

#### 4.2.1. Gremlin

While the data discussed previously illustrate how the epithelial cancer cells themselves are affected by their own aberrant expression of BMP antagonists, the following studies outline situations in which aberrant BMP antagonist production could affect the epithelial cells in a paracrine fashion. A study that adapted a genomic approach was among the first studies supporting a possible contribution of stromal secreted BMP antagonists in cancer progression [46]. To determine which factors are expressed differently by stromal cells in the basal cell carcinoma (BCC) microenvironment and normal skin, gene expression profiles were generated from primary stromal cell cultures. *GREM1* was not only found to be upregulated by stromal cells isolated from BCC, but also for a number of other cancer types such as prostate, colon, pancreas and esophageal cancer [46]. These antagonists produced and secreted by the stromal cells could possibly support cell growth and inhibit both differentiation and apoptosis.

While the upregulation of Gremlin suggests a possible involvement in the pathology of a disease, further evidence is required to draw conclusions about a causative or supporting role. Two studies have investigated the prognostic significance of stromal Gremlin expression in cancer progression [47,48]. Stromal Gremlin expression in colorectal cancer, as determined by *GREM1* in situ hybridization, was found to be associated with a less advanced cancer stage, decreased lymphovascular invasion and improved recurrence-free and overall survival [47]. However, in breast cancer the opposite has been reported. There Gremlin expression was found to predict worse clinical outcomes [48]. The beneficial prognostic traits associated with stromal Gremlin expression in colorectal cancer seems counterintuitive considering the association found between stromal Gremlin expression and induction of EMT as implicated in generating cancer stem cells (CSC) and the development of metastatic cancer [49,50].

Interestingly, multiple studies have shown that Gremlin expression is causally associated with both the presence and maintenance of mesenchymal characteristics, not only during development but also in cancer stem cell niches [50,53,64,65]. In one particular study, Gremlin and α-SMA expressing CAFs in colorectal cancers were observed near the tumor invasive front where tumor cells showed nuclear accumulation of β-catenin and the loss of the tight junction protein occludin [66]. Gremlin expression was found to be associated with the occurrence of carcinoma cells with an EMT phenotype near the invasive front. Additional in vitro experiments in which CRC cell lines were stimulated with Gremlin showed indications of EMT as defined by downregulation of E-cadherin, and increased Snail and N-cadherin expression [66]. It is well known that TGF-β, often found to be upregulated in the tumor stroma, can induce EMT and that BMP signaling could oppose this process [67]. These results together support the existence of a paracrine interaction between CAFs and cancer cells in which Gremlin could be involved in tumor progression, either by shaping the microenvironment to support the tumor cells or by facilitating processes such as EMT.

#### 4.2.2. Noggin

Less convincing roles are found for fibroblast-derived Noggin. However, one study reported that xenografts of a prostate cancer cell line (LNCaP) upregulated *Nog* in the stromal compartment of mice overexpressing Shh using species specific primers [68]. While not showing a direct causal relationship, this study does show that cancer cells could have the ability to instruct the stroma to produce certain BMP antagonists, which in turn could favor tumor growth.

Expression of BMP antagonists by the tumor stroma could partially explain the conflicting data concerning the role of BMP antagonists in cancer. Could the downregulation of BMP antagonists, often observed in human cancer samples, be regulated by stromal cells to halt cancer cell proliferation? Is this normalizing cue finally misused by the continuously evolving cancer cells later in tumor progression (possibly due to mutations in the BMP pathway) to facilitate tumor growth? In the light of the CAF heterogeneity, it would be valuable to investigate which CAF subset(s) are the main producers of BMP antagonists. Could it be the “tumor restricting” CAF-populations trying to slow down tumor progression or the “tumor promoting” CAFs instructed by the cancerous cells? The steady increase in the amount of research conducted on CAFs might in the future provide us an answer that will also help us understand why a favorable prognosis was reported in some studies, but a poor prognosis was found in others.

### 4.3. The Endothelial Cells

In normal tissue endothelial cells are found in a quiescent state but tumor growth and its dissemination are heavily dependent on tumor vascularization [69]. If not sufficiently formed this can slow down the rate by which the tumor grows and progresses [69]. The “angiogenic switch” is defined as the moment in which there is a transition in the vasculature from a quiescent state to a proliferative state, thereby inducing angiogenesis [70]. This process has been shown to be promoted and influenced by the recruitment of innate immune cells (discussed later) and CAFs [70].

#### Gremlin

Apart from functioning as a BMP antagonist, Gremlin has been shown to have its own intrinsic signaling pathway that is BMP ligand independent. Recombinant Gremlin was found to bind with high affinity to endothelial cells in vitro, activating the intracellular signaling pathways extracellular-signal-regulated kinase (ERK), paxillin and focal adhesion kinase (FAK), which regulate migration and matrix remodeling by endothelial cells [71]. This resulted in increased invasion through collagen and fibrin gels, but also initiated neovascularization in vivo in the chorioallantoic membrane of the chick embryo [71]. Additionally, Gremlin was found to be accumulated on lung cancer-associated endothelial cells compared to a normal lung vasculature [71]. These findings were later found to be caused by binding of Gremlin to the VEGF receptor 2 [72,73]. Interestingly, monomeric Gremlin showed the opposite effect by acting as a VEGF receptor 2 antagonist [74]. These results suggest that dimeric Gremlin could directly and BMP independently support tumor growth by promoting the “angiogenic switch” facilitating the generation of an endothelial network to provide the cancerous cells with oxygen and a route by which they can metastasize. This could also explain the tumor-promoting effects observed in studies utilizing overexpression models with supraphysiologic levels of Gremlin. Interestingly, if Gremlin is indeed causally involved in increased angiogenesis within tumors, monomeric Gremlin could be a novel therapeutic strategy to prevent neo-vascularization. This was recently demonstrated in a study showing that monomeric Gremlin had an inhibiting effect on the angiogenic and tumorigenic potential of murine prostate and breast cancer cells in vivo [74].

### 4.4. The Immune Cells

Immune cells comprise an important part of the tumor microenvironment influencing all stages of cancer development and progression. Since many cells in the microenvironment respond to BMP signaling, one would expect this for immune cells as well. Indeed, BMP signaling is found to be implicated in CD4 T-cell homeostasis and activation [75,76], natural killer (NK) cell development [77], chemotaxis of monocytes [78] and activation of programmed death-ligand 1 (PD-L1) and PD-L2 expression by dendritic cells [79,80]. Unfortunately, there is hardly any data on how BMP antagonists affect the immune cells in the tumor microenvironment. In other, mostly inflammatory, diseases such as fibrosis, arthritis and atherosclerosis, studies have been performed with potential implications for cancer as well [81]. Below we will discuss these studies in more detail.

#### 4.4.1. Gremlin

Multiple studies have described that binding of Gremlin to the VEGF receptor 2 on endothelial cells, next to inducing angiogenesis, also evokes a proinflammatory response that leads to the induction of several chemokines and cell adhesion molecules. Consequently, increased leukocyte adhesion and extravasation is observed [82,83]. These authors also showed in a mouse xenograft experiment that the presence of Gremlin expressing MCF7 breast cancer cells caused a significant increase of CD45^+^ cells, consisting of primarily F4/80^+^ macrophages, compared to mock-transfected MCF7 cells [83]. Besides inducing a proinflammatory response in endothelial cells, a recent study showed that Gremlin activates the Notch pathway that is linked to renal inflammation in chronic kidney disease [84]. If Gremlin can indeed provoke a proinflammatory response that promotes the influx of F4/80^+^ cells, the outcome would be highly dependent on the subtype of macrophage that is being recruited. While the M1-macrophage is considered to exert favorable pro-inflammatory behavior, the M2-macrophage is thought to be anti-inflammatory and could prevent an anti-tumor immune reaction. However, data from two other studies suggest that Gremlin functions as an inhibitor of monocyte/macrophage attraction [85,86]. In conclusion, more research is needed to better understand the effect of Gremlin on immune cells in the tumor.

#### 4.4.2. Noggin

The involvement of Noggin in immunomodulation is even less well studied with only a few articles reporting an effect on immune cells. In a study on rheumatoid arthritis, the researchers showed in the methylated bovine serum albumin (mBSA)-induced arthritis mouse model that Noggin haploinsufficient (*Noggin^+/LacZ^*) mice had an increased number of CD4^+^ lymphocytes in their synovial fluid compared to wild type mice [87]. Noggin was additionally shown to decrease the expression of inflammatory factors in the vascular wall of mice from the diabetic *db/db* mouse model often used for atherosclerosis research [88]. If Noggin can indeed counteract the recruitment of CD4 T-cells and lower the expression of inflammatory factors, this would mean that Noggin counteracts inflammation. Noggin probably exerts its effects by binding BMPs since BMP2 and BMP4 are implicated in being involved in vascular inflammation [88]. The suppression of an inflammatory reaction is an undesirable property in established cancers, as an inflammatory response against cancer cells has been shown to predict positive clinical outcomes in solid tumors [89].

## 5. Conclusions

With multiple studies reporting opposing effects of BMP antagonists, clearly much remains to be deciphered and we still do not fully understand their multifaceted effects. Despite the increasing awareness for the role of the BMP pathway in oncogenesis, very little research has been conducted on understanding how the different cell types within the tumor contribute to this complex signaling interplay. In this review we have discussed several studies that demonstrate a role for BMP antagonists in the microenvironment in addition to their effect on the cancer cells. Unfortunately, many of these results seem largely circumstantial. More research is needed to truly understand how BMPs and BMP antagonists carry out their effects. We hypothesize that the effect is coherent with a (non-) functional BMP signaling pathway within the cancer cells and that these cancer cells instruct their microenvironment to secrete factors, either BMPs or BMP antagonists, to allow tumor growth. A better understating of the role of BMP antagonists in cancer progression would be valuable as it could potentially provide novel therapeutic strategies for many cancer types.

## Figures and Tables

**Table 1:** Overview of studies concerning the role of BMP antagonists in cancer.

Antagonist	Model Used	Organ	Finding	Effect on Tumor	References
**Chordin**	Ovarian carcinoma cell lines BG1 and PEO14 and human specimens	Ovaries	Chordin downregulated in tumors and cancer cell lines	Suppressing	Moll et al. 2006 [33]
**Gremlin**	Basal cell carcinomas	Skin	Increased tumor cell proliferation	Promoting	Sneddon et al. 2006 [46]
	Human specimens	Various	Increased *GREM1* expression was found in uterine, ovary, lung, kidney, colon, pancreas and breast carcinomas	Promoting	Namkoong et al. 2006 [51]
	Clear cell renal cell carcinomas	Kidney	*GREM1* promoter methylation correlates with increased malignancy and active angiogenesis	Suppressing	van Vlodrop et al. 2010 [52]
	Human hereditary mixed polyposis (HMPS) syndrome specimens	Intestines	Mutation drives *GREM1* expression	Promoting	Jaeger et al. 2012 [24]
	Primary mesothelioma cells	Mesothelium	shRNA mediated downregulation of *GREM1* inhibits cell growth and renders susceptibility to paclitaxel	Promoting	Tamminem et al. 2013 [53]
	Pancreatic neuroendocrine tumors	Pancreas	High Gremlin expression identified as a favorable prognostic marker	Suppressing	Chen et al. 2013 [54]
	Primary Glioma cell lines	Brain	Increased proliferation and progression from cell cycle arrest	Promoting	Yan et al. 2014 [55]
	Human mesothelioma cell lines with Gremlin overexpression	Mesothelium	Overexpression increases tumor vascularization with an increased chance to metastasize	Promoting	Yin et al. 2017 [56]
	4T1 mouse mammary tumor model, breast cancer cell lines 66cl4 and 67NR and human specimens	Breast	*GREM1* associated with metastases and worse prognosis in estrogen receptor-negative breast cancer patients	Promoting	Neckmann et al. 2019 [57]
**PRDC**	Human endometrial cancer (EC) specimens and Ishikawa and HEC-1A EC cell lines	Endometrium	*PRDC* was found to be downregulated in ECs and inhibited proliferation of cancer cells	Suppressing	Tsubamoto et al. 2016 [37]
	*In silico* analysis on gastric cancer microarray expression data and in vitro and in vivo experiments with the gastric cancer cell line MNK-45 with a PRDC downregulation	Stomach	*PRDC* was found to be upregulated in gastric cancer. Silencing of *PRDC* in MNK-45 resulted in vitro in decreased proliferation, migration and invasion. in vivo, tumor formation and lymph node metastasis was inhibited.	Promoting	Ran et al. 2019 [38]
**Noggin**	Noggin-overexpression mouse	Intestines	De novo crypt and polyp formation	Promoting	Haramis et al. 2004 [18]
	Osteolytic PC-3 cancer cells with Noggin overexpression	Prostate	Inhibition of PC-3 cell expansion	Suppressing	Feeley et al. 2006 [26]
	B16-F1 melanoma cell line growth in the chick embryonic optic cup	Skin	Decreased invasive growth	Suppressing	Busch et al. 2007 [58]
	Melanoma cell lines	Skin	Expression of Noggin renders the cells less/non-susceptible for BMP7-growth inhibiting effects	Promoting	Hsu et al. 2008 [59]
	A549 non-small cell lung cancer cell line with Noggin overexpression	Lung	Growth inhibition	Suppressing	Feeley et al. 2009 [27]
	Keratin14-driven Noggin overexpression mouse	Skin	Development of spontaneous hair-follicle derived tumors	Promoting	Sharov et al. 2009 [60]
	Osteolytic PC-3 cancer cells retrovirally transduced with Noggin	Prostate	Decreased bone loss and smaller tumors	Suppressing	Virk et al. 2009 [61]
	Osteolytic PC-3 cancer cells transduced with Noggin-shRNA	Prostate	Decreased growth of cancer cells	Promoting	Secondini et al. 2011 [62]
	Breast cancer cell lines and human specimens	Breast	Increased expression in metastatic lesions and Noggin overexpression facilitates bone colonization	Promoting	Tarragona et al. 2012 [63]
**Sclerostin**	Human non-small cell lung cancer specimens and cell lines	Lung	SOSTDC1 expression associated with a better survival and reduced cell proliferation of cell lines	Suppressing	Liu et al. 2016 [35]
	Human gastric cancer specimens	Intestines	Lower *SOSTDC1* expression in primary tumors compared to normal tissue and in metastases compared to primary tumors.	Suppressing	Cui et al. 2019 [34]
